# A Low Cost Fe_3_O_4_–Activated Biochar Electrode Sensor by Resource Utilization of Excess Sludge for Detecting Tetrabromobisphenol A

**DOI:** 10.3390/mi13010115

**Published:** 2022-01-11

**Authors:** Suxing Luo, Meizhi Yang, Yuanhui Wu, Jiang Li, Jun Qin, Feng Feng

**Affiliations:** 1 Department of Chemistry and Chemical Engineering, Zunyi Normal College, Zunyi 563006, China; 2 College of Chemistry and Environmental Engineering, Shanxi Datong University, Datong 037009, China; possumlee@126.com (J.L.); qj187@hotmail.com (J.Q.); feng-feng64@263.net (F.F.); 3 Office of Academic Research, Guizhou Open University, Guiyang 550023, China; yangmeizhi@mail.gyig.ac.cn

**Keywords:** sludge-based biochar, Fe_3_O_4_, TBBPA, electrochemical sensor

## Abstract

Owing to its ubiquity in natural water systems and the high toxicity of its accumulation in the human body, it is essential to develop simple and low-cost electrochemical sensors for the determination of 3,3′,5,5′-tetrabromobisphenol A (TBBPA). In this work, Fe_3_O_4_–activated biochar, which is based on excess sludge, was prepared and characterized using scanning electron microscopy (SEM), energy dispersive spectroscopy (EDS), Fourier transform infrared spectroscopy (FTIR) and BET analysis to analyze its basic features. Subsequently, it was used to fabricate an electrochemical sensor for the detection of TBBPA. The electrochemical test results revealed that the Fe_3_O_4_–activated biochar film exhibited a larger active surface area, a lower charge transfer resistance and a higher accumulation efficiency toward TBBPA. Consequently, the peak current of TBBPA was significantly enhanced on the surface of the Fe_3_O_4_–activated biochar. The TBBPA sensing platform developed using the Fe_3_O_4_–activated biochar composite film, with relatively a lower detection limit (3.2 nM) and a wider linear range (5–1000 nM), was successfully utilized to determine TBBPA levels in water samples. In summary, the effective application of Fe_3_O_4_–activated biochar provided eco-friendly and sustainable materials for the development of a desirable high-sensitivity sensor for TBBPA detection.

## 1. Introduction

Tetrabromobisphenol A (TBBPA) is a kind of brominated flame retardant [1], which has been widely produced and globally used in electronics, textiles, epoxy resins and padding materials [2]. TBBPA will be released into the environment if the wastes of these products are abandoned or inappropriately treated. Moreover, exposure to TBBPA could seriously threaten human health by adversely affecting multiple cell types [3] and leading to endocrine disorders [4]. Thus, it is of the utmost importance to establish a low-cost, simple operation with responsive, sensitive methods to detect TBBPA.

As of now, lots of methodologies and processes have been implemented for determining TBBPA levels, such as electrochemical methods [5], high-performance liquid chromatography [6], gas chromatography-mass spectrometry [7] and liquid chromatography-mass spectrometry [8]. Among the above-mentioned techniques, the electrochemical method has been becoming the most highly enticing for sensor research. Until now, many electrochemical sensors have been developed for the detection of TBBPA, such as polypyrrole-AuNPs-MWCNTs [5], g-C_3_N_4_/GCE [9], Au NPs/SH-beta-CD/GO [10] and MWCNTs–Fe_3_O_4_ [11]. These prepared sensors can detect TBBPA sensitively, but expensive carbonaceous materials (CNTs, GO, C_3_N_4_) or noble metals (Au nanoparticles) narrowed their widespread application due to their high cost. To address these problems, it is highly desirable to exploit earth-abundant electrode materials for electrochemical TBBPA sensing application.

Biochar has gained noticeable popularity due to its excellent adsorption properties, its low cost and easy preparation. Biochar can be synthesized using crops, poultry droppings and excess sludge from the major residue of municipal sewage treatment plants. Recently, biochar has gradually attracted attention as an effective adsorbent [12] and as an electrode modifier [13,14,15,16,17]. For example, Sant’Anna et al. [15] reported an electrochemical sensor based on biochar and reduced graphene oxide, which was applied to detect carbendazim in real samples with a limit of quantification (LOQ) of 7.7 nmol/L. Oliveira et al. [16] constructed an electrochemical sensor with activated biochar for the spontaneous preconcentration of methyl parathion and for further quantitative determination in drinking water. In this work, cheap sludge was used for preparing biochar. Meanwhile, to increase surface functional groups and perform well, the derived biochar is necessary for its further treatment by HNO_3_. In addition, metal oxide nanoparticles [18,19,20,21] exhibit outstanding conductivity and facilitate electron transfer [22]. In this study, we propose a novel and eco–friendly electrochemical sensor based on Fe_3_O_4_–activated biochar for the sensitive detection of TBBPA with low cost. The thus-prepared Fe_3_O_4_–activated biochar sensor could be used for detecting TBBPA in water samples for practical applications.

## 2. Materials and Methods

### 2.1. Materials and Reagents

The excess sludge was collected from the Gaoqiao Wastewater Treatment Plant in Zunyi, China. Ferrous chloride, ferric chloride (≥99.9%) and tetrabromobisphenol A were purchased from Aladdin Industrial Corporation (Ontario, CA, USA). Nafion (5 wt%) was purchased from Shanghai Format New Energy Tech. Co., Ltd. (Shanghai, China). All other chemicals were of analytical grade and were used without further purification.

### 2.2. Preparation of Fe_3_O_4_-activated Biochar

**Preparation of sludge-based biochar.** Generally, the surface area and pore size of biochar increases with rising pyrolysis temperatures [23]. However, higher temperatures could result in the porous structure being destroyed and blocked [24] and, more importantly, higher pyrolysis temperatures would decrease the number of functional groups [25]. Therefore, 600 °C was carefully chosen as the pyrolysis temperature for biochar preparation [26] in this study. Excess sludge was firstly dried overnight at 105 °C, and then pyrolyzed at 600 °C for 3 h in a tubal furnace under a N_2_ atmosphere. However, the high ash content in the sludge-based biochar is disadvantageous to chemical sensors due to its poor electrical conductivity. Therefore, the resulting product was then immersed in 0.5 M HF and evaporated at 100 °C in the fume hood [27] to generate ashless biochar. Finally, the obtained solid product was washed with distilled water to a neutral pH and then dried at 105 °C to generate sludge-based biochar.

**Activation of sludge-based biochar.** Typically, 0.2 g of sludge-based biochar was added to 50 mL of 50% (*v*/*v*) nitric acid solution in a refluxing system under 60 °C, lasting for 3 h, to obtain activated biochar, and then washed to neutral and dried.

**Preparation of Fe_3_O_4_–activated biochar.** Firstly, 0.1 g of activated biochar was dispersed in 50 mL of deionized water. Then, 0.002 mol of FeCl_3_ and 0.001 mol of FeCl_2_ were dissolved in 10 mL of deionized water together, then added dropwise into the activated sludge biochar suspension while stirring vigorously. Subsequently, ammonia was added dropwise until the pH of the suspension increased to 10.0, and then the suspension was heated at 80 °C for 3 h. Finally, the dispersion was separated easily by an external magnet and washed with distilled water. After drying in vacuum at 60 °C, the Fe_3_O_4_–activated biochar was successfully synthesized and could be used as a modifier agent with electrodes.

### 2.3. Characterization Measurements

The morphology of the Fe_3_O_4_–activated biochar was observed by scanning electron microscopy (SEM, ZEISS EVO 15) with energy dispersive spectrometry (EDS) to analyze elemental composition. The specific surface area and pore size of the sludge–based biochar were determined using a BSD–PS (Beishide Instruments, Beijing, China). An FTIR analyzer (VERTEX70 spectrometer, Bruker Co., Bremen, Germany) was used for analyzing the surface functional groups of the pulverized samples. Electrochemical impedance spectroscopy (EIS) was employed to characterize different the kinds of electrodes using a 2273 electrochemical system (Princeton Applied Research PARSTAT2273 Electrochemical Workstation).

### 2.4. Electrode’s Construction and Detection

A glassy carbon electrode (GC) with a diameter of 3 mm was cleaned successively with 0.3 μM and 0.05 μM alumina slurries on an abrasive paper, ultrasonicated for several minutes in ethanol and deionized water respectively, and dried in air. Fe_3_O_4_–activated biochar was ultrasonically dispersed in an aqueous solution (containing 0.2 wt% Nafion) to obtain 1 mg/mL Fe_3_O_4_–activated biochar suspension. Then, the electrode was modified using the suspension through dropping and drying on the electrode surface. Electrochemical experiments were carried out using a 2273 electrochemical system (Princeton Applied Research PARSTAT2273 Electrochemical Workstation) with a conventional three–electrode system. A bare or modified glassy carbon electrodewas used as the working electrode. Meanwhile, the counter and reference electrodes were used a platinum wire and an Ag/AgCl electrode respectively. In addition, all experiments below were performed at room temperature. Three repeats for each sample (*n* = 3) were conducted in the electrochemical measurements.

## 3. Results and Discussion

### 3.1. Characterization of Synthesized Materials

**Figure 1:**
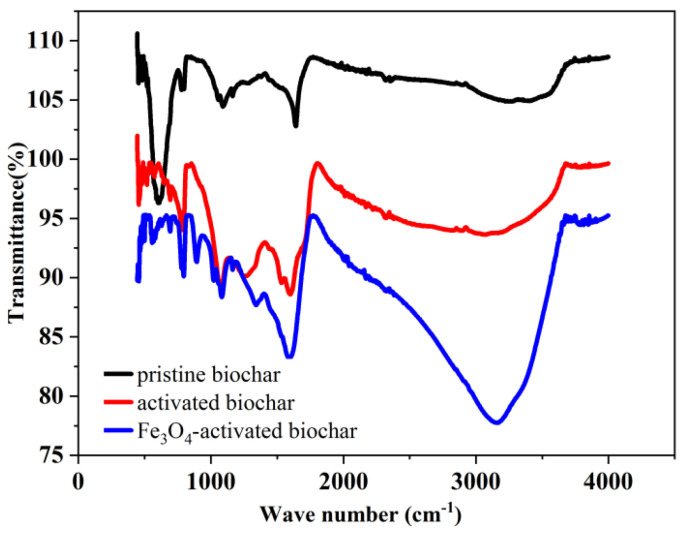
FTIR spectrums performed for thus–repared samples.

In order to reveal the functional groups of the thus-prepared Fe_3_O_4_–activated biochar, FTIR analysis was performed. As shown in Figure 1, the peaks present at 3410 and 1649 cm^−1^ are attributed to –OH groups. The slightly weak peak at 1092 cm^−1^ corresponded to the C-OH stretching vibration band. Furthermore, a characteristic band of deformation vibrations outside the plane of the C-H groups in the aromatic structures was at 673 cm^−1^ [28]. Noticeably, after the activation, new functional groups appeared compared to the pristine sludge-based biochar. The new peaks at 1542 cm^−1^ and 1287 cm^−1^ corresponded to the presence of nitro groups, suggesting that the nitration and oxidation reaction may be co-occurring [29]. However, the peak at 894 cm^−1^ was only detected on the spectrum of the Fe_3_O_4_–activated biochar, which most likely was assigned to the Fe-O stretch of Fe_3_O_4_. Additionally, the peak intensity of the Fe_3_O_4_–activated biochar was stronger than the pristine biochar and the activated biochar under identical conditions, indicating that the Fe_3_O_4_–activated possessed larger amounts of functional groups.

**Figure 2:**
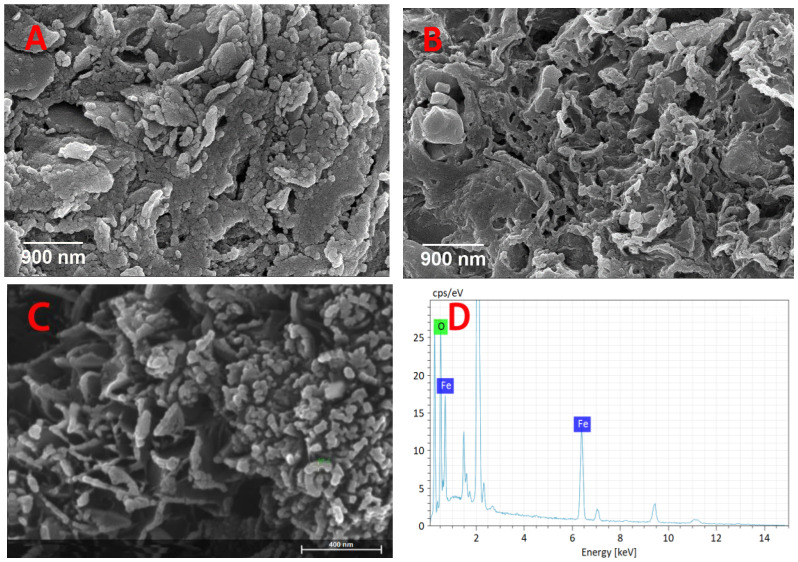
SEM images of the (**A**) pristine biochar, (**B**) activated biochar and (**C**) Fe_3_O_4_–activated biochar. (**D**) EDX elemental analysis of the Fe_3_O_4_–activated biochar.

To characterize the detailed morphology of the prepared samples, SEM was employed to identify the structural characteristics (Figure 2). The pristine biochar presented a plate–like structure, consisting of dozens of irregular block–based particles (Figure 2A), while the surface morphology of the activated biochar, as shown in Figure 2B, had a relatively rough structure with irregular cavities. Some works in the literature reported that the activation resulted in the formation of functional groups on the walls or edges of opening pores [30,31]. According to Figure 2C, a large number of irregular Fe_3_O_4_ particles were distributed on the surface of the magnetic sludge biochar. The SEM-EDX results (Figure 2D) confirmed the existence of Fe species, and the weight ratio of iron on the surface of the Fe_3_O_4_–activated biochar was detected as 53.98 wt%, indicating that Fe_3_O_4_ exists on the surface of the activated biochar.

**Figure 3:**
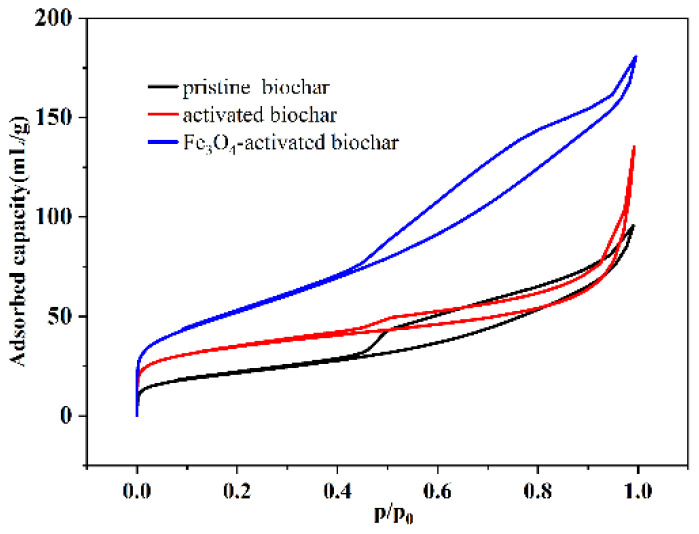
N_2_ adsorption–desorption isotherm characterizations of Fe_3_O_4_–activated biochar.

The thus–prepared samples were further characterized using the N_2_ adsorption–desorption curve. As depicted in Figure 3, the isotherm curves were consistent with the IUPAC classification type Ⅱ curve and H3 type hysteresis loop. The specific surface areas of the pristine biochar, activated biochar and Fe_3_O_4_–activated biochar were calculated as 75.34, 126.27 and 247.45 m^2^/g, respectively. Compared with the pristine biochar, the specific surface area of the activated biochar increased sharply, because a porous structure with a large number of functional groups (such as carboxyl groups) could readily adsorb a large amount of Fe^2+^/Fe^3+^. Consequently, a large amount of Fe_3_O_4_ particles was synthesized and coated on the surface of the biochar to further increase the specific surface area.

### 3.2. EIS Characterization of Fe_3_O_4_–Activated Biochar/GC

**Figure 4:**
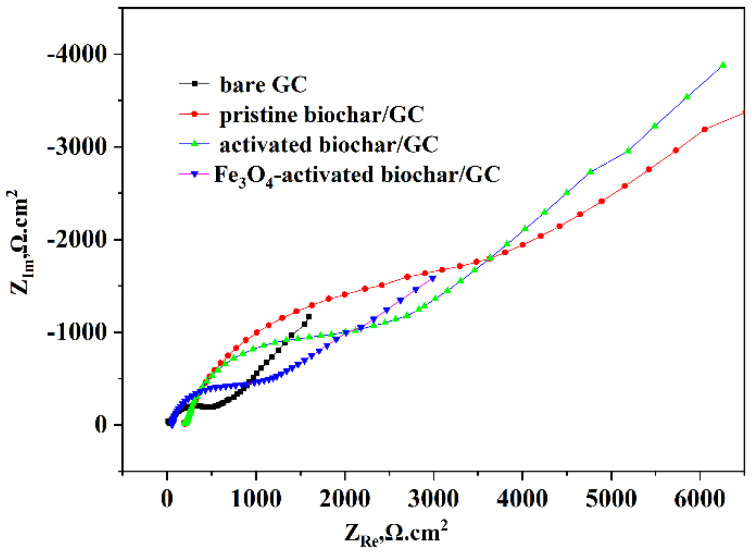
EIS of various electrodes.

Electrochemical impedance spectroscopy (EIS) is an effective tool with which to characterize the interface properties of electrochemical sensors. Figure 4 shows the impedance spectra, represented as Nyquist plots (ZIm vs. ZRe), for (a) bare GC (b) pristine biochar/GC (c) activated biochar/GC and (d) Fe_3_O_4_–activated biochar/GC in 5 mM Fe(CN)_6_^3−^/^4−^ containing a 0.1 M KCl solution (pH = 7.0). All of them show the impedance plots comprising depressed semicircles and straight–line features. Generally, the diameter of the semicircle is directly proportional to the electro transfer resistance (Rct) in the Nyquist plots of EIS [32]. The bare GC (curve (a)) had a relatively small electron transfer resistance (Rct), and after being modified with the pristine biochar, the significant increase in the Rct indicated that the pristine biochar has a stronger obstruction effect than the bare GC. After the activated biochar had been immobilized on the electrode surface, the Rct had a large electron transfer resistance with a big semicircle domain, and this can be attributed to the presence of more functional groups (carboxyl, nitro) on its surface, which carried a negative charge. Thus, the electron transfer was retarded between the redox probe and the double electrochemical layer. However, the Rct dramatically decreasedwhen modified the Fe_3_O_4_–activated biochar, which implied that Fe_3_O_4_ modification could effectively accelerate the electron transfer as we expected. The results demonstrated that the electrochemical sensor was well fabricated.

### 3.3. Electrochemical Behavior of TBPPA on Fe_3_O_4_–Activated Biochar/GC

**Figure 5:**
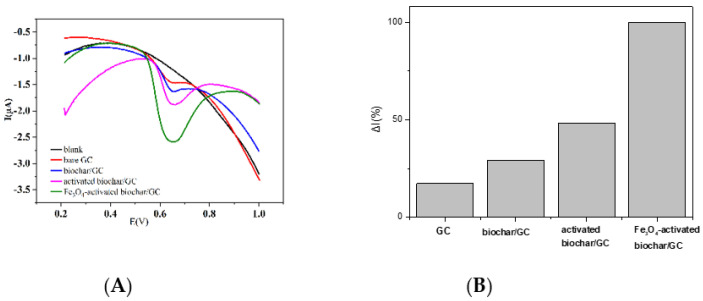
(**A**) Differential pulse voltammograms obtained for various electrodes in the presence of 0.5 μmol/L of TBPPA, BR buffer (pH 4.0). (**B**) Relative ΔI_P_ intensities for tested electrodes obtained from A.

The voltametric behaviors of TBPPA were investigated under differential pulse voltammogram conditions using bare GC, pristine biochar/GC, activated biochar/GC and Fe_3_O_4_–activated biochar/GC. As shown in Figure 5, all the electrodes studied had an anodic peak at 0.65 V identified as TBPPA oxidation, but relatively poorly defined oxidation peaks were observed for the bare GC and biochar/GC. Compared with the other three electrodes, the peak current increased clearly for the Fe_3_O_4_–activated biochar/GC. The oxidation and this improvement of the voltametric response could be attributed to its larger specific surface area and more surface functional groups due to the contribution of further modification. Possible interaction mechanisms between the organic molecule and the surface of Fe_3_O_4_–activated biochar can mainly be summarized in three ways [13,15,16,33]: (1) the larger specific surface area leads to stronger physical adsorption, (2) π–π type interaction could occur between the aromatic ring of the organic molecule and biochar aromatic structures, and (3) the hydroxyl groups of TBPPA could interact with the carboxyl groups of the biochar to formation hydrogen bonds. Moreover, the mechanisms described above might work together in different combinations. Thus, the proposed electrochemical sensor, Fe_3_O_4_–activated biochar/GC, had a synergic effect on the signal intensity, which provided an anodic peak current that was nearly six–fold higher than those observed using bare GC, as shown in Figure 5B.

### 3.4. Optimization of Experimental Parameters

**Figure 6:**
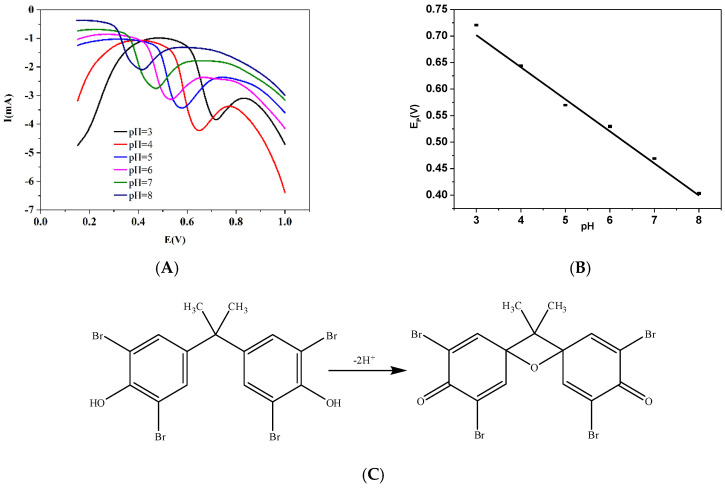

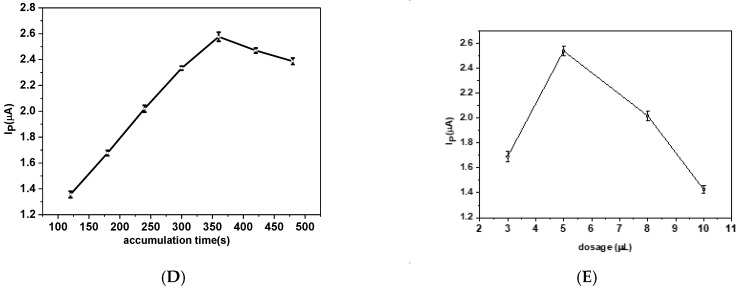
(**A**) Differential pulse voltammograms obtained using Fe_3_O_4_–activated biochar/GC for the pH study in the presence of 5.0 μmol/L TBPPA in BR buffer (pH 3.0 to 8.0). (**B**) Correlation of Ep versus pH variation. (**C**) The possible oxidation reaction mechanism of TBPPA. (**D**) The effects of accumulation time on the peak current. (**E**) The effects of dosage on the peak current.

To develop an appropriate analytical condition for the sensitive detection of TBPPA, experimental parameters were optimized. Firstly, the pH influence on the electrochemical behavior of TBPPA was evaluated by using a BR buffer solution with a pH range of 3.0–8.0. As shown in Figure 6A, it was clear that the DPV signals reached a maximum at a pH of 4.0, and then decreased as the pH increased further. Thus, we chose a pH of 4.0 as the optimal pH value. Moreover, the potential of the oxidation peak shifted to less positive values as the pH increased, which illustrated that H^+^ was directly involved in the electrochemical oxidation process of TBBPA. Furthermore, the DPV signal of TBPPA showed a linear relationship between the pH and the anodic peak potential (Ep) with an equation of Ep (V) = 0.893–0.0060 pH (Figure 6B). Importantly, the slope value was approximately close to the theoretical value of 0.059 V per pH (298 K), indicating that there was an equal number in the proton and electron transfer in the electrochemical oxidation process of TBBPA. According to the results of previous studies, the possible electrochemical oxidation mechanism, if the number of electrons and protons involved in the TBBPA oxidation process stayed equal, could involve a 2H^+^/2e^−^ transfer, as suggested in Figure 6C. In this mechanism, TBBPA is oxidized to quinone [10,11].

In general, open–circuit accumulation is a simple but effective way to increase the test sensitivity of electrochemical determination. In addition, the accumulation time could directly affect the adsorption capacity of TBBPA at the electrode. Therefore, the influence of the accumulation time, at ranges from 120 to 480 s, on the oxidation of TBBPA by the Fe_3_O_4_–activated biochar/GC was also investigated. Consequently, the current of TBBPA tended to rise with the increase in the accumulation time, and the maximum current was obtained at 360 s (Figure 6D). Thus, 360 s was chosen as the optimal accumulation time, implying the saturated adsorption of TBBPA at the electrode surface occurred in this time.

Lastly, the effect of the modified volume of the Fe_3_O_4_–activated biochar biochar on the DPV response was investigated in the range of 3 to 10 μL. The DPV responses for TBPPA increased with the rising of the dose from 3 to 5 μL, but decreased when the dosage exceeded 5 μL (Figure 6E), probably owing to the agglomeration of Fe_3_O_4_ nanoparticles and the poor conductivity of the thick film. Thus, 5 μL of 1 mg/mL Fe_3_O_4_–activated biochar suspension was used to modify the GC.

### 3.5. Electrochemical Determination of TBPPA

**Figure 7:**
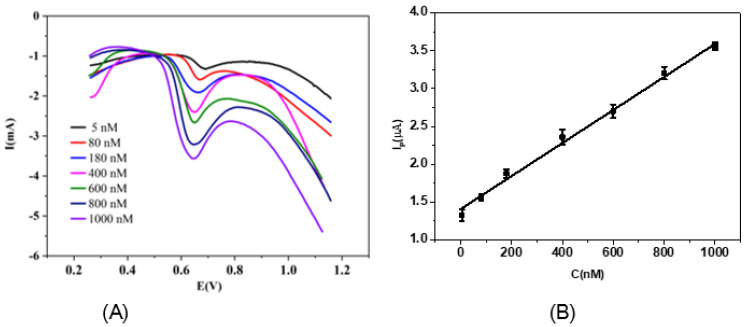
(**A**) DPV responses for different concentrations of TBPPA on Fe_3_O_4_–activated biochar/GC. (a–g) 5, 80, 180, 400, 600, 800 and 1000 nM, respectively. (**B**) Calibration curve.

As shown in Figure 7A, it was revealed that the oxidation peak currents gradually increased with the growing TBPPA concentration from 5 to 1000 nM. Meanwhile, a linear relationship between the peak current and the concentration of TBPPA was obtained with a linear regression of I_P_ (µA) = 1.413 + 0.0022C (nM) (Figure 7B). The detection limit of the fabricated sensor was calculated to be as low as 3.2 nM. In addition, some analytical results of the TBPPA determination, using both other comparative electrochemical sensors and this work, are listed in Table 1. According to these results, the fabricated sensor presented a lower detection limit compared to most of the other electrochemical sensors with affordable raw materials. Thus, it would be very competitive at determining TBPPA levels.

### 3.6. Reproducibility, Interferences and Real Samples Analysis

We measured and compared the oxidation peak currents for 50 nM TBBPA for five successive determinations to determine the reproducibility of the Fe_3_O_4_–activated biochar/GC. The relative standard deviation (RSD) was calculated to be 2.76% among five measurements with the same sensor, showcasing the excellent repeatability and detection precision of the biosensor under continuous operation. In addition, the fabricated sensor retained 92.13% (*n* = 3) of its initial response for 50 nM TBBPA after being stored in the refrigerator at 4 °C for 14 days, confirming the good stability of the sensor.

To explore the potential of Fe_3_O_4_–activated biochar/GC for the detection of TBPPA in real-life applications, the fabricated sensor was used to detect TBPPA in water samples. Water samples were collected from the Guizhou Chishui river basin (Guizhou, China). The samples were passed through a 0.22 μm filter membrane to remove impurities. After filtration, a 100 mL water sample was concentrated to 10 mL through vacuum evaporation, then diluted with BR buffer and the pH value was adjusted to 4.0. After 360 s of open-circuit accumulation, differential pulse voltammograms from 0.25 to 1.1 V were recorded for TBPPA with a 50 mV pulse amplitude and a 50 ms pulse width. The oxidation peak currents at about 0.65 V were measured as the analytical signal for TBBPA. It was continuously measured five times, and the RSD of the measurement results were calculated. As TBPPA concentrations were not detected in these samples, the standard addition method was used, and these results are displayed in Table 2 and satisfactory results were obtained, with the recoveries between 92.7 ± 1.2% and 99.1 ± 2.3%.

## 4. Conclusions

In this study, a sensitive, simple fabrication and cost-effective electrochemical sensor (an Fe_3_O_4_–activated biochar) was successfully developed based on excess sludge. The proposed sensor showed excellent electrochemical performance towards TBBPA sensing when compared to precursor materials separately, and may be applied to other applications. However, the presented activated biochar was prepared at a relatively high temperature, and it is still a big challenge to optimize the synthesis process for a mild and low energy consuming condition. As further studies are conducted to prepare biochar with a special structure and abundant functional groups, we believe that Fe_3_O_4_–activated biochar may be actually applied for the efficient detection of TBPPA, and more significantly, might be a promising method for the sensitive detection of TBPPA in environment monitoring.

## Figures and Tables

**Table 1:** Comparisons of Various Electrochemical Sensors for TBPPA.

Type of the Electrode	Linear Range (nM)	Detection Limit (nM)	Reference
CNTs@ZIF-67/CP	10–1500	4.23	[34]
CTAB/NG-TPA/GCE	10–1000	9.0	[35]
g-C_3_N_4_/GCE	20–1000	5.0	[9]
AuNPs-PSSA	0.1–10 nM	0.025	[36]
BDD electrode	50–10,000 M	27	[37]
SH-β-CD-AuNPs/GO/GCE	15–7000	1.2	[10]
TOMA/GCE	1.84–919	1.05	[38]
AB/GCE	18.4–643	11.2	[38]
Fe_3_O_4_–activated biochar/GC	5–1000	3.2	This work

Note: CNTs@ZIF-67: carbon nanotubes@ zeolitic imidazole framework–67; CTAB/NG–TPA: hexadecyl trimethyl ammonium bromide/nitrogen-doped graphene-1, 3, 6, 8-pyrenetetrasulfonic acid; g-C_3_N_4_: graphitic carbon nitride; AuNPs-PSSA: gold nanoparticles (AuNPs) and poly(sulfosalicylic acid) (PSSA) composite film; BDD: boron-doped diamond; SH-β-CD-AuNPs-1/GO/GCE: gold nanoparticles (AuNPs) –thio–β–cyclodextrin(SH-β–CD)/graphene oxide; TOMA: trioctadecyl methylammonium bromide; AB: acetylene black.

**Table 2:** Determination of TBPPA in Real Samples (*n* = 3).

Samples	Original	Added (nM)	This Method (*n* = 3)		
			Found	Recovery (%)	RSD
1	0	100	99.1	99.1 ± 2.3	4.4
2	0	200	189.2	94.6 ± 2.6	3.5
3	0	400	381.2	95.3 ± 1.7	4.1
4	0	600	556.2	92.7 ± 1.2	4.7

Considering the co-existence of contaminants in water samples, the influence of interferences on TBBPA quantification was evaluated. The results showed that the inorganic species, including Ca^2+^, Mg^2+^, Fe^3+^, NO_3_^−^ and SO_4_^2−^, in 50-fold concentrations almost had no influence on the detection of 100, 200, 400 or 600 nM TBPPA, with deviations below 5%. Moreover, five-fold amounts of some organic substances, such as bisphenol A, bisphenol, and carbamazepine, showed no significant influence on the DPV signal, with deviations below 5%.
